# Scientific collaboration for a better, more sustainable tomorrow

**DOI:** 10.1093/nsr/nwab035

**Published:** 2021-03-25

**Authors:** H N Cheng, Angela K Wilson, Luis Echegoyen

**Affiliations:** The American Chemical Society, USA; John A. Hannah Distinguished Professor, Department of Chemistry, Michigan State University, USA; Robert A. Welch Chair Professor of Chemistry, Department of Chemistry and Biochemistry, University of Texas at El Paso, USA

Collaboration among scientists and the chemistry community is as much a feature of the research process as the desire to discover. At the American Chemical Society (ACS), encouraging international scientific collaboration between chemists has long been a key component of our global outreach and an objective for our organization as a whole. Indeed, the stated mission of ACS is ‘Advancing the broader chemistry enterprise and its practitioners for the benefit of Earth and all its people.’

This mission cannot be achieved by working solely amongst ACS members, even with our over 155 000 members living and working in over 100 countries around the world, including a significant contingent in the Asia-Pacific region. Cooperation with other chemical societies around the world is also key to advancing the chemistry enterprise and enriching the lives of chemists, chemical engineers and other allied professionals in academia, industry and government agencies. This effort is solidified through numerous formal and informal partnerships supporting the chemistry enterprise with organizations including the Chinese Chemical Society (CCS), the Federation of Asian Chemical Societies (FACS), the European Chemical Society (ECS), the Latin American Federation of Chemical Associations (FLAQ) and many others.

In China, ACS has had the opportunity to work directly with the Chinese chemistry community through the CCS in a variety of ways, including our past participation in the multilateral Chemical Sciences & Society Summit (CS3). The CS3 program addressed some of the leading global challenges of our time and explored ways chemistry can contribute solutions. Much like the values of CS3, the UN Sustainable Development Goals (SDGs) are a pathway to channel scientific collaboration to solve some of the most pressing challenges of our time: climate change, global health and more. Although US participation in the CS3 program ended in 2018, ACS remains an ardent champion of the SDGs and multilateral international research collaboration, and we look forward to addressing these challenges with our colleagues and partners throughout the globe.

ACS has also been fortunate to collaborate with the Chinese chemistry community on a key priority for both of our nations: instilling a love of the chemical sciences at an early age to train the next generation of scientists. Promoting education programs focused on science, technology, engineering and math (STEM) must be a continuing universal effort of the global chemistry enterprise, and toward that end we have many opportunities to work together in this space. To provide an example, ACS and CCS have worked together on the ACS Chemistry Festival program. Hosted in collaboration with our ACS International Chapters in China, the Chemistry Festival in Dalian in 2016 was a success, enabling hundreds of children in the region to experience a hands-on learning opportunity and the joy of chemistry.

The COVID-19 pandemic has only further underlined the importance of scientists coming together. When scientists reach across national borders, we all benefit. Multiple studies have confirmed that transnational collaborative research yields great returns: higher citations, sharing of resources, expertise, and it brings researchers closer together. In our careers, international collaboration in research has been especially significant. As members of the ACS Presidential succession, we are familiar with the many contributions of chemists and chemical engineers from around the globe, especially from our work at various US organizations in academia, industry and government.

In addition to being home to some of the leading chemists of our time, the United States and China represent the world's largest economies and producers of scientific output. As we consider the many challenges that lie ahead, we are certain of one critical aspect: we cannot move forward alone. As leaders in this field, we have a joint responsibility to encourage the multilateral scientific community to find ways to collaborate, encourage candid conversations to protect scientific integrity, and pose solutions to some of our greatest challenges, including those highlighted by the SDGs. Harnessing innovation to tackle inequality in education, water sanitation, renewable energy, chemistry for health and climate change are just a few of the ways we can help lead the way.

While the past year has created much uncertainty across the globe, we look forward to a time in the near future when we will be able to come together again with our partners from around the globe to collaborate in person. Despite these difficulties, we will continue to seek ways to support the community worldwide and work together to benefit the global chemistry enterprise. We face many challenges ahead and we at ACS are steadfast in our belief that chemistry is a vital part of the solution.

